# Mucins Differently Expressed in Various Ampullary Adenocarcinomas

**DOI:** 10.1186/1746-1596-6-102

**Published:** 2011-10-25

**Authors:** Tao Wang, Ye M Liang, Peng Hu, Yu F Cheng

**Affiliations:** 1 Department of Radiation Oncology, QiLu Hospital of Shandong University, West Wen hua Rd 107, Jian, Shandong, 250012, China

**Keywords:** mucin, ampulla of Vater, common bile duct, neoplasm staging

## Abstract

**Background:**

We investigated the occurrence and clinical significance of mucin expression in ampullary adenocarcinoma.

**Methods:**

We retrospectively analyzed clinical, pathological, and survival data from 74 ampullary adenocarcinoma patients who received radical operation from January 2004 to November 2006.

**Results:**

The tumors were located in the lower end of the common bile duct (46%), papillary duodenum (42%), and ampullary duodenum (12%), and expressed MUC1 (72%), MUC2 (20%), MUC5AC (43%), and MUC6 (27%). Expression of MUC1 was associated with tumor differentiation (OR: 4.71, 95% CI: 1.26, 17.66, P = 0.021). Expression of MUC5AC was associated with age (OR: 1.07, 95% CI: 1.11, 1.14, P = 0.026) and less vessel invasion(OR: 0.14, 95% CI: 0.03, 0.72, P = 0.019). The survival rates were not significantly different when patients had or had no expression of MUC1, MUC2, MUC5AC, or MUC6 in tumor. Patients with tumors positive for MUC5AC in the papillary duodenum had worse survival than those with tumors negative for MUC5AC (P = 0.044).

**Conclusions:**

Expression of MUC1 was high (72%) in ampullary adenocarcinoma, while expressions of MUC2, MUC5AC, and MUC6 were lower. Mucins are useful markers to diagnose and identify ampullary adenocarcinoma, particularly in determining the degree of malignancy of ampullary adenocarcinoma.

## Background

Ampullary adenocarcinoma, which occurs in the area of the head of the pancreas, comprises about 5-6% of cancers arising in that region [1]. Cancer of the ampulla is a rare disease, occurring in less than one per 100,000 cases [2]. Because even small lesions in the ampulla can obstruct the bile duct, early jaundice and early presentation occurs, which may lead to the overall five-year survival of 30% to 50%, which is better than that of other periampullary neoplasms. Two major histological types of these tumors have been recognized, which are an intestinal type that arises from intestinal mucosa of the papilla, and a biliopancreatic type that arises from the biliopancreatic ductal epithelium [3]. These two types have been separated by immunohistochemically analyzing their expression of MUC2 [4], which is one of a class of macromolecular glycoproteins with a complex glycosylation structure. The histological differentiation is a key factor in the prognosis of ampullary cancer [2]. The intestinal subtype is negative for MUC1 and the biliopancreatic subtype is negative for MUC2 [2,5-7]. The differing expression of mucins in ampullary adenocarcinomas is attributed to the development of histogenetically differing carcinomas that come from the intestinal or the pancreaticobiliary mucosa.

Mucins are high molecular weight glycoproteins that are distributed on the cell surface, where they serve as selective barriers, and participate in intercellular recognition, adhesion, and signal transduction [8]. Mucins are widely distributed in gastrointestinal tumors and normal tissues, of which MUC1, MUC2, MUC5AC, and MUC6 are more important [9]. Abnormal mucin expression is associated with tumors, where mucins play an important role in occurrence and development [8]. Various mucins express differently in tumors and normal tissues. For example, normal pancreatic tissue mainly expresses MUC1 and MUC6, while ampullary adenocarcinoma mainly expresses MUC1 and MUC5AC [10], along with MUC6 and MUC2 [3,11,12]. Combined detection of mucins has been used to diagnose and differentiate pancreatic ductal adenocarcinoma and ampullary adenocarcinoma [11-15].

Ampullary carcinoma prognosis is currently based on stage, lymph node metastases, negative surgical margins, and presence of microsatellite instability, with stage being the only independent prognostic factor, and mucin expression not predicting survival [3]. Circulating antibodies to MUC1 are associated with a favorable prognosis in pancreatic cancer [16], while expression of MUC1 indicates a poor prognosis in pancreatobiliary pancreatic head tumors [17]. The expression of MUC5AC is associated with better prognosis for ampullary carcinoma [18], and with better survival of invasive ductal carcinoma of the pancreas [19].

This study explored the relationships between diagnosis, identification, and survival of ampullary adenocarcinoma and the expressions of MUC1, MUC2, MUC5AC, and MUC6. The positive expression of MUC1 was the highest of the four mucins investigated, and the expressions of MUC1 and MUC5AC were associated with levels of tumor aggressiveness. Patients with tumors positive for MUC5AC in the papillary duodenum had worse survival.

## Subjects and Methods

### Subjects

This retrospective study enrolled 74 patients from Qilu Hospital of Shandong University from January 2004 to November 2006 who were pathologically diagnosed with ampullary adenocarcinoma, received radical operation, and had complete clinical, pathological, and survival data available. This study was approved by the ethical review board of Qilu Hospital, and informed consent was waived because of its retrospective nature.

### Materials

The solution of 10% goat serum and primary antibody against MUC6 were obtained from Boster Biotech (Wuhan, Hubei, China). Primary antibodies against MUC1, MUC2, and MUC5AC were obtained from the Laboratory of Genetics, School of Life Sciences, Fudan University (Shanghai, P.R. China). The goat anti-rabbit secondary antibody, the StreptAvidin-Biotin Complex (SABC) immunohistochemical assay kit, and the DAB chromogenic agent were obtained from Boster Biotech. The Leica CM1900 microtome was from Wetzlar, Germany, and the BH2 light microscope was from Olympus (Tokyo, Japan).

### Methods

After surgery, the resected tumor sample was sent to the department of pathology, where samples were fixed in neural formaldehyde and preserved in paraffin until studied, when they were sliced by microtome and then deparaffinized by immersing sections twice for 12 minutes in xylene. The sections were hydrated by immersing them twice for 10 minutes in absolute ethanol, then twice for 5 minutes in 95% ethanol, then 5 minutes in 70% ethanol, and finally 5 minutes of rinsing with water. Endogenous peroxidase was inactivated by immersing sections in 3% hydrogen peroxide for 10 minutes and then immersing the sections in PBS twice for 5 minutes each time. Next, antigens were retrieved by twice immersing the samples in citrate buffer, boiling the immersed sections in a microwave oven for 10 seconds, and allowing the sections to cool to room temperature. Then, the sections were immersed in PBS twice for 5 minutes each time.

The sections were blocked by incubating them in 10% goat serum at 37°C for 30 minutes, and then incubated with primary MUC antibodies diluted 1:25 or with PBS (control group) overnight at 4°C. Then, the sections were rinsed twice in PBS for 5 minutes each time. Then, the sections were incubated with goat anti-rabbit secondary antibodies diluted 1:100 at 37°C for 30 minutes and then rinsed three times with PBS for 5 minutes. The sections were incubated with SABC complex diluted 1:100 at 37°C for 30 minutes, and then washed three times in PBS for 5 minutes each wash. Color was developed by adding diaminobenzidine (DAB), and, when positive staining was observed under a light microscope without nonspecific background staining, the sections were washed with water for 5 minutes. The sections were then counterstained by immersing them in hematoxylin for 30 minutes and then washing them with water for 5 to 10 minutes. Then, the sections were dehydrated with 70% ethanol for two minutes, followed by xylene for two minutes.

Gross changes were observed at low magnification of 10 x, and positive cells were counted at a high magnification of 40 x. Positive cells were scored as: 0, negative; 1, 1% to 33%; 2, 34% to 67%; and 3, >67%. Positive cells had brown granules in the nucleus and cytoplasm, and staining intensity was scored as: 0, no staining; 1, light yellow; 2, brownish yellow; and 3, brown in over half of the observed area. The semi-quantitative analysis of cells was the sum of both scores, with the final scores indicating: 0, negative; 1 to 2, +; 3 to 4, ++; and 5 to 6, +++. The scoring was performed by two pathologists unaware of the study, and discrepancies were resolved by consensus.

### Statistical analysis

Because the cases were unevenly distributed among the histological grades, we clustered them into two categories for statistical analysis: one category was undifferentiated (G4) and poorly differentiated (G3) cases, and the other was moderately (G2) and well differentiated (G1) cases. Logistic regression analysis analyzed the odds ratio of significant factors associated with patients with positive expression of each MUC family (MUC1, MUC2, MUC5CA, and MUC6). Survival time was measured from the time of operation to the time of death or the last date of follow-up. The survival rates of patients with positive and negative expression of MUC family were compared through a Kaplan-Meier analysis with log-rank test that measured survival over time. The statistical assessments were all two-sided, and were considered significant if *P *< 0.05. Statistical analyses were performed using SPSS 15.0 statistics software (SPSS Inc., Chicago, IL).

## Results

### Patient and tumor information

The ampullary adenocarcinoma patients in this study included 46 men (62.2%) and 28 women (37.8%) whose mean age was 60.4 ± 11.7 years (range 27 to 80 years) (Table 1). The tumors were located in the lower end of the common bile duct in 34 cases (46%), the papillary duodenum in 31 cases (42%), and the ampullary duodenum in 9 cases (12%). The mean tumor size was 2.08 ± 1.12 cm. Of the 74 patients, 71 underwent pancreatoduodenectomy, one underwent pylorus-preserving pancreatoduodenectomy, and two underwent local tumor resection. No perioperative deaths occurred. Five cases of postoperative pancreatic leakage occurred, three of gastroparesis, and six of delayed wound healing. All complications were cured by conservative therapy.

**Table 1:** Patients' demographics and baseline characteristics

Variable	No. (%) (N = 74)
Age (years)	60.36 ± 11.72
Gender	
Male	46 (62.2)
Female	28 (37.8)
Tumor size (cm)	2.08 ± 1.12
Location	
Lower end of the common bile duct	34 (45.9)
Papillary duodenum	31 (41.9)
Ampullary duodenum	9 (12.2)
Tumor differentiation	
No & Low	39 (52.7)
Middle & High	35 (47.3)
Tumor classification	
T0 & T1 & T2	38 (51.4)
T3 & T4	36 (48.6)
Node classification	
N0	45 (60.8)
N1	29 (39.2)
Distant metastasis	
M0	71 (95.9)
M1	3 (4.1)
TNM stage	
0 & I	28 (37.8)
II & III & IV	46 (62.2)
Vessel invasion	19 (25.7)
Nerve invasion	19 (25.7)

All the patients were followed through June 30, 2008, and 30 patients had died (41%). Most deaths were due to tumor recurrence, liver metastasis, or extensive metastases in the abdominal and pelvic cavity.

### Expression of mucins in ampullary adenocarcinoma

Expression of MUC1 was detected in 38 of 53 cases (72%), MUC2 in 10 of 50 cases (20%), MUC5AC in 22 of 51 cases (43%), and MUC6 in 13 of 48 cases (27%) (Table 2). Most MUC1-positive tumors were poorly differentiated or undifferentiated, but most MUC1-negative tumors were well-differentiated or moderately differentiated. MUC5AC-positive tumors seldom had blood vessel invasion. Logistic regression analysis found that expression of MUC1 was not significantly correlated with TNM stage or presence of blood vessel and nerve invasion of tumors but significant association was found between MUC1 expression and tumors with middle or high levels of differentiation(OR: 4.71, 95% CI: 1.26, 17.66, P = 0.021). MUC5AC expression was significantly associated with age (OR: 1.07, 95% CI: 1.11, 1.14, P = 0.026), and negative correlations between MUC5AC expression and vessel invasion (OR: 0.14, 95% C.I.: 0.03, 0.72, P = 0.019) was noticed. Expression of MUC2 and MUC6 were not significantly correlated with any index of tumors. In addition, there was no association between the expression of each mucin gene and the location of the tumors.

**Table 2:** MUC gene expression and logistic regression analysis of associations between MUC family genes and clinical characteristics

	MUC1 (n = 53)	MUC2 (n = 50)	MUC5AC (n = 51)	MUC6 (n = 48)
	
Variable	No. (%)			
Gene Expression				
Positive	38 (71.7)	10 (20.0)	22 (43.1)	13 (27.1)
Negative	15 (28.3)	40 (80.0)	29 (56.9)	35 (72.9)

		OR (95% C.I.)		

Age	1.02 (0.97, 1.07)	1.01 (0.95, 1.07)	1.07 (1.11, 1.14)*	1.04 (0.97, 1.10)
Gender				
Female	1.00	1.00	1.00	1.00
Male	2.45 (0.73, 8.42)	2.96 (0.56, 15.73)	0.88 (0.28, 2.74)	2.22 (0.52, 9.54)
Tumor size	0.90 (0.50, 1.65)	0.72 (0.32, 1.58)	1.60 (0.86, 2.97)	0.50 (0.22, 1.11)
Location				
Lower end of the common bile duct	1.00	1.00	1.00	
Ampullary duodenum	0.22 (0.02, 1.93)	-	1.08 (0.13, 8.80)	-
Papillary duodenum	0.35 (0.10, 1.31)	1.25 (0.31, 5.09)	0.58 (0.18, 1.91)	1.50 (0.41, 5.51)
Tumor differentiation				
No & Low	1.00	1.00	1.00	1.00
Middle & High	4.71 (1.26, 17.66)*	0.91 (0.22, 3.62)	1.55 (0.51, 4.74)	0.47 (0.13, 1.72)
Tumor classification				
T0 & T1 & T2	1.00	1.00	1.00	1.00
T3 & T4	1.50 (0.45, 5.05)	0.43 (0.10, 1.90)	2.05 (0.66, 6.31)	1.56 (0.43, 5.59)
Node classification				
N0	1.00	1.00	1.00	1.00
N1	1.09 (0.32, 3.69)	1.00 (0.23, 4.11)	1.90 (0.61, 5.90)	0.36 (0.08, 1.52)
Distant metastasis				
M0	1.00	1.00	1.00	1.00
M1	0.38 (0.02, 6.47)	-	1.33 (0.80, 22.57)	-
TNM stage				
0 & I	1.00	1.00	1.00	1.00
II & III & IV	1.28 (0.37, 4.39)	0.54 (0.13, 2.18)	1.88 (0.57, 6.21)	0.69 (0.19, 2.50)
Vessel invasion,				
No	1.00	1.00	1.00	1.00
Yes	1.63 (0.38, 6.92)	1.13 (0.25, 5.17)	0.14 (0.03, 0.72)*	0.46 (0.09,
Nerve invasion				
No	1.00	1.00	1.00	1.00
Yes	1.63 (0.38, 6.92)	1.29 (0.68, 5.94)	3.32 (0.92, 12.01)	0.36, 6.19)

* indicates statistical significance where *P *< 0.05.

### Mucin expression and survival of patients

**Figure 1:**
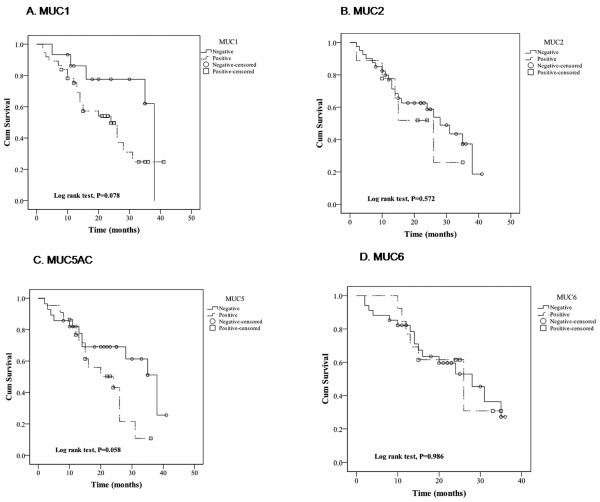
**Survival curves of the expression of MUC family genes**. Kaplan-Meier curves analyze the relationships between survival and expression of MUC1 (A), MUC2 (B), MUC5AC (C), and MUC6 (D), where those patients with tumors negative for the gene expression are shown with solid lines, and those with tumors positive for the gene expression are shown with dashed lines. The open circles (○) indicate censored patients with tumors negative for the gene expression, and the open squares (□) indicate censored patients with tumors positive for the gene expression.

Survival curves are shown in Figure 1. Survival rates were not significantly affected by expression of MUC1, MUC2, MUC5AC, or MUC6, though lack of MUC1 or MUC5AC expression trended toward improved survival compared to tumors that did express MUC1 or MUC5AC (*P *= 0.078 for MUC1 and *P *= 0.058 for MUC5AC) (Figure 1).

**Figure 2:**
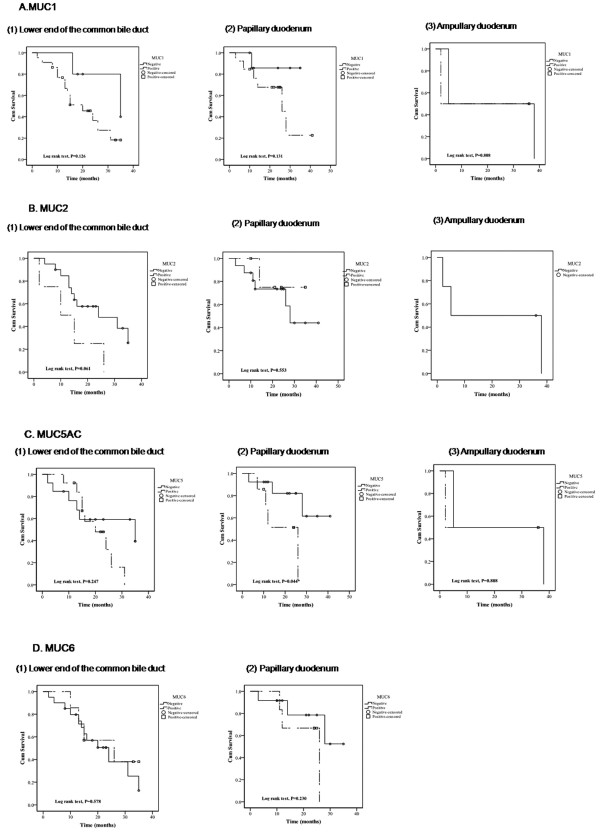
**Survival curves of the expression of MUC family genes by different tumor location**. Kaplan Meier curves analyze the relationships between survival and location of tumors for MUC1 (A), MUC2 (B), MUC5AC (C), and MUC6 (D), for tumors located in (1) the lower end of the common bile duct, (2) the papillary duodenum, and (3) the ampullary duodenum. The solid lines indicate tumors that were negative for the MUC gene expression, the dashed lines indicate tumors that were positive for the MUC gene expression, the open circles (○) indicate censored patients with tumors negative for the gene expression, and the open square (□) indicate censored patients with tumors positive for the gene expression.

We compared survival curves based on the locations of the tumors, which were in the lower end of the common bile duct, in the papillary duodenum, or in the ampullary duodenum (Figure 2). Among patients with tumors in the papillary duodenum, those with detectable MUC5AC had worse survival than those without detectable MUC5AC (Figure 2, *P *= 0.044). No other significant associations between tumor location and MUC gene expression were detected, though expression of MUC2 in tumors in the lower end of the common bile duct trended toward worse survival than no MUC2 in tumors there (*P *= 0.061).

**Figure 3:**
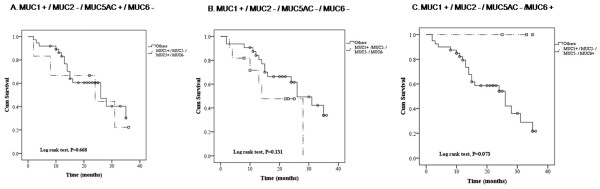
**Survival curves of the expression of combinations of Mucins (n = 44)**. Kaplan-Meier curves analyze the relationships between survival and combinations of expression of MUC genes, including the combination of (A) MUC1 +, MUC2 -, MUC5AC +, and MUC6 -; (B) MUC1 +, MUC2 -, MUC5AC -, and MUC6 -; and (C) MUC1 +, MUC2 -, MUC5AC -, and MUC6+ indicated by dashed lines and other combinations indicated by solid lines.

We further analyzed the survival curves of the 44 patients who had all four MUC isoforms tested. We did not find significantly different survival rates for those having the combinations of being positive for MCU5AC and negative for MUC6, negative for MUC5AC and MUC6, or negative for MUC5AC and positive for MUC6AC (1+/2-/5+/6-, p = 0.668; 1+/2-/5-/6-, p = 0.131; 1+/2-/5-/6+, p = 0.073)(Figure 3).

## Discussion

The expression of MUC1 occurred more frequently than the other mucins studied, and it was associated with a higher degree of differentiation of ampullary adenocarcinoma. This result concurs with findings that MUC1 is related to malignant behavior of pancreatic mucinous cystic tumors [20]. Expression of MUC1 may affect patient prognosis by inhibiting the formation of the E cadherin and β-catenin complex, which would decrease intercellular adhesion and promote the invasion and metastasis of tumors [21], by enhancing tumor cell invasiveness through the involvement of MUC1 in the cell signal transduction pathway that is controlled by platelet-derived growth factor β [22], and by increasing the resistance of tumor cells to cytotoxic chemotherapeutics like 5FU [23].

The expression of MUC5AC was associated with worse survival among patients who had tumors in the papillary duodenum than those with tumors with undetectable MUC5AC. Additionally, MUC5AC expression was associated with patient age and negatively correlated with vessel invasion. These results are in agreement with findings that MUC5AC expression levels decreased as the malignancy of gallbladder tumor [24] and gastric signet ring cell carcinoma [25] increased; colon cancer negative for MUC5AC had more tumor recurrence, metastasis, and worse prognosis than those positive for MUC5AC [26]; and pancreatic ductal adenocarcinoma positive for MUC5AC expression was correlated with better prognosis [19] and less vascular invasion. In a separate study, we found that MUC5AC was significantly associated with TNM stage of pancreatic ductal adenocarcinoma and its vascular invasion than those negative for MUC5AC expression (unpublished data). The expression of MUC5AC may be related to the vascular invasiveness of tumors because MUC5AC is a gastric-type secretory mucin that can enhance intercellular adhesion by promoting the formation of a high-viscosity gel [27].

Our study has explored the connection between periampullary tumors and mucins, which is slightly different than the connection between pancreatic cancer and mucins. Our institution routinely stains some gastrointestinal tumors for mucins. Further understanding the expression and mechanisms of mucins may help to improve survival of periampullary tumors. Our study is limited by the small number of cases studied and by the incomplete analyses of the tumors for the four MUC genes studied. We independently analyzed the expression of each MUC gene by logistical regression analysis (Table 2), but only a few patients had more than one MUC gene expressed, which was too small of a sample to statistically analyze. The small sample size did not allow us to find any statistical significance as we sought to test the hypothesis that mucin expression patterns may differentiate the prognosis for a distinct tumor type or stage. Future work will benefit from larger numbers of patients studied and more complete analyses of MUC genes.

In summary, ampullary adenocarcinoma patients may benefit from detecting the composition of mucins in resected tumor specimens, since this will facilitate both diagnosis and differential diagnosis. Mucin composition analysis may also help to determine the degree of malignancy of ampullary adenocarcinoma.

## Competing interests

No competing interests.

## Authors' contributions

YML designed the study and wrote the protocol, TW performed research and wrote the first draft of the manuscript. PH manged the literature searches and analyses. YFC contributed important reagents. All authors read and approved the final manuscript.
